# Patient-reported outcomes on gastrointestinal tolerance and adherence to a pea protein plant-based enteral formula in children and adults

**DOI:** 10.3389/fnut.2025.1619884

**Published:** 2025-12-17

**Authors:** Stanley A. Cohen, Vanessa Millovich, Dwan Newman, Nicole Withrow, Lucille Beseler, Christina J. Valentine

**Affiliations:** 1 Children's Center for Digestive Health Care, Morehouse School of Medicine, Atlanta, GA, United States; 2 Kate Farms, Inc, Goleta, CA, United States; 3 Family Nutrition Center of South Florida, Boca Raton, FL, United States

**Keywords:** enteral nutrition, yellow pea protein based formula, fiber, vegetarian, gastrointestinal tolerance, vegan, nutrition support, formula intolerance

## Abstract

**Introduction:**

Enteral Nutrition through a feeding tube or orally can improve patient outcomes when tolerated to achieve nutritional requirements. While experts have provided feeding guidelines to enhance safety and efficacy, challenges in gastrointestinal (GI) tolerance such as diarrhea, constipation, bloating and vomiting often complicate adherence. Problems such as malnutrition, morbidity, and mortality occur when gastrointestinal intolerance results in the provision of formulas stopping and starting, and therefore delays, in optimal intake. Formulas vary widely in their composition, including differences in the primary protein source, degree of protein hydrolysis, inclusion of common allergens, fiber content, use of artificial or nonnutritive sweeteners, and use of artificial colors. A distinctive yellow pea protein, plant-based enteral formula (PPPBF) that includes fiber, and is free from common allergens and artificial and nonnutritive sweeteners may improve tolerance and adherence.

**Methods:**

We examined the patient or caregiver reports of gastrointestinal (GI) intolerance and adherence in both pediatric and adult participants using two electronic surveys. The initial survey compared pre and during utilization of the PPPBF and the second survey, sent 12 months later, evaluated the same reported outcomes, specifically on the use of a PPPBF.

**Results:**

There were 392 respondents to the initial survey [n = 160 Pediatric (< /=19 years); and *n* = 232 Adults (>/=20 years)]. A positive trend toward GI tolerance and adherence was observed.

**Conclusion:**

A PPPBF with fiber may promote enteral tolerance and support nutritional intake. If used as a first-choice formulation option, it may reduce formula switching that often results from GI intolerance.

## Introduction

Enteral nutrition (EN) is essential for individuals who depend solely on a liquid or reconstituted medical food/formula delivered as tube feedings or as oral intake (1, 2). EN can serve as a sole source of nutrition, function as a meal replacement, or simply provide additional nutritional support. There are published guidelines to promote safe practices and delivery (3); however, there are no consistent standards of practice for clinicians to consider as they choose formulas for their patients (4). Not having a targeted approach to using EN often results in a minimum of 2–3 formula changes prior to achieving tolerance (4). A major consequence of these switches is that a person can be on inadequate nutritional intake for days, weeks and even months before finding a formula that they tolerate (5). Starting and stopping EN delays optimal nutrient intake which can lead to malnutrition or worsen the condition of an already malnourished patient (6). Data suggests that 1/3 of patients admitted to the hospital are malnourished (5) or become malnourished while in medical facilities (6) which impacts health outcomes and mortality (7) and puts a burden on the healthcare system and overall costs of care (8, 9). While disease-related malnutrition is well established as a primary factor in the incidence of malnutrition, a major contributor can be intolerability of the formula regimen (5). In indicated cases, enteral nutrition improves clinical outcomes and is one important proactive strategy to mitigate the risks associated with malnutrition (10). It has been reported that only 5.2% of malnourished hospitalized adult patients in the United States are receiving EN even though it can improve nutritional status (7). IgE mediated, non-IgE mediated and mixed cow's milk allergy (CMA) are common in children and adults that can lead to formula intolerance and decreased adherence (11).

CMA is one of the most common IgE food allergies in early life with an estimated prevalence in developed countries of up to 3% at 1 year of age. In adults, prevalence is lower with estimates around 0.5% (12). However, US population-based surveys reveal this can be much higher, finding 1.9% of the US population (children and adults) having CMA with convincing IgE-mediated symptoms and 4.7% of the US population report that they have a CMA (13). Variability in reported prevalence rates may be attributable to the diagnostic challenges associated with non-IgE-mediated CMA. The rate of IgE-mediated CMA decreases while non-IgE-mediated and mixed CMA increases with increasing age (11). The prevalence of all 3 types of CMA may contribute to intolerance on dairy-based formulas.

To more rigorously characterize patterns of enteral formula utilization, gastrointestinal intolerance, adherence, and formula-transition practices, a survey was administered to pediatric and adult individuals receiving enteral nutrition. The objective was to evaluate differences in tolerance and use patterns between previously prescribed enteral nutrition (EN) formulas and a pea protein, plant-based enteral formula (PPPBF).

## Materials and methods

A cross-sectional cohort survey study was performed September of 2019 to understand the patient or patient caregiver experience, usage, and their reported outcomes before using and while using a PPPBF. To gather additional insight into reported usage and health related outcomes, a follow-up survey was sent 12-months later to those who completed the 1st survey. The study protocols were approved by a centralized Institutional Review Board (IRB), Integ Review, Protocols 002 and 003, respectively.

Both surveys were electronic, administered utilizing REDCap^®^ and sent via email. REDCap is a validated research database developed by Vanderbilt University to collect and store information (14). The initial survey (Supplementary Figure 1) consisted of 32-items and was sent to patients and/or the caregivers of patients who had previously requested a sample of a PPPBF (Standard 1.0, Peptide 1.5, Pediatric Standard 1.2, or Pediatric Peptide 1.5, Komplete) manufactured by Kate Farms, Inc. Potential participants were identified for inclusion through an online database (Kate Farms, Inc, Goleta CA). Participants who completed the entire survey were provided with an electronically sent $25 Visa gift card for their time and participation. Data were collected on demographic characteristics, reported general health, formula use characteristics, and outcomes on gastrointestinal (GI) symptoms referred to as intolerance, and adherence. Adherence was defined as being able to consume at least 75% of the amount recommended by their health care provider (HCP) for the formula previously used and for the time when the PPPBF was used. All data was de-identified and stored in password protected and locked data sets. 12 months later, the follow-up survey (Supplementary Figure 1) was sent to those who had completed the 1st survey and consisted of 35-items, including the same questions on demographic characteristics, reported general health, formula use characteristics, and outcomes of GI tolerance and adherence while on the PPPBF. Participants who completed the entire survey were provided with an electronically sent $25 Visa gift card for their time and participation. All data was again de-identified and stored in password protected and locked data sets.

### Statistical analysis

The number of respondents who reported use of at least one PPPBF and who completed the surveys constituted the sample size. Descriptive statistics were assessed with frequencies and percentages due to the categorical nature of all data. A chi-square test was employed to determine if there were significant differences in the frequency of reported symptoms on the PPPBF. A *post-hoc* analysis divided participants into two age groups: pediatric (ages 1–19 years) and adult (ages 20 years and older). Data were then stratified by these groups and compared using chi-square tests, which were also used to identify potential confounders. Null responses were retained and included in the final analysis, and all statistical analyses and plots were generated using R, version 3.6.1.

## Results

### Participant demographics

The 1st survey was emailed to the list of eligible participants (*n* = 1,592). There were 398 respondents, of which 392 completed all 32 items of the questionnaire, and were included in the final analysis. When stratified by age, the pediatric cohort (PC) of 159 included 87 (22.1%) who were 1–5 years of age (y); 42 (10.7 %) who were 6–12 y; and 30 (7.6 %) between 13 and 19 y. The adult cohort (AC) of those ≥20 years of age (*n* = 233; 59.2%) had a majority, *n* = 111 (28.2%), in the 20–40 y age bracket, with 65 (16.5%) between 41 and 60 y and 57 (14.5%) over 60 y. Females comprised 225 (57.3%) of the total respondents (Table 1). Significantly more participants in the 20–40 y age group identified as self-reporters, when compared to the participants in the >60 y age group (*p* = 0.005).

**Table 1:** Participant demographics (*n* = 392).

**Variable**	**1st survey *n* = 392 n (%)**	**2nd survey *n* = 213 *n* (%)**
**Age**		
1–5 years	87 (22.1)	49 (23)
6–12 years	42 (10.7)	32 (15)
13–19 years	30 (7.6)	16 (7.5)
20–40 years	111 (28.2)	65 (30.5)
41–60 years	65 (16.5)	24 (11.3)
>60 years	57 (14.5)	26 (12.2)
Prefer not to answer	1 (0.3)	1 (0.5)
**Gender**		
Female	225 (57.3)	124 (58.2)
Male	165 (42.0)	86 (40.4)
Prefer not to answer	3 (0.8)	3 (1.4)

The second survey was distributed to the participants who had completed the first survey, resulting in 213 completed responses. Due to the small group size, subsequent analyses were conducted on the entire cohort of respondents. Age was determined not to be a confounder, so results were not reported based on the initial age stratifications. Demographic information can be reviewed in Table 1. Due to small group sizes, subsequent analyses were conducted on the group level.

### Use characteristics of enteral formula users

Participants were asked to describe how the formula was used each day and were able to “check all that apply” meaning that they were able to state if the formula was used via a feeding pump and/or by mouth. In the initial survey, more than half answered that the formula was used by mouth (*n* = 211, 53.7%) and 32.8% (*n* = 129) used the formula via pump. The responses were similar in the 2^nd^ survey, where more than half answered that the formula was used by mouth (*n* = 116, 54.5%) and 34.7% (*n* = 74) using a formula pump (Table 2).

**Table 2:** Comparison of enteral formula use characteristics between first and second surveys.

**Variable**	**1st survey *n* = 392 *n* (%)**	**2nd survey *n* = 213 *n* (%)**
**Administration route**		
**(participants could select more than 1 option)**		
Mouth	210 (53.6)	116 (54.4)
Syringe	59 (15.1)	33 (15.5)
Gravity Bag	37 (9.4)	18 (8.5)
Feeding Pump	129 (32.9)	74 (34.7)
**Estimated % of nutrition from PPPBF**		
< 50%	105 (26.8)	70 (32.9)
50–90%	128 (32.7)	69 (32.4)
>90%	150 (38.3)	63 (29.6)
Unsure	8 (2.0)	10 (4.7)
**Length of time on PPPBF**		
< 1 months	30 (7.7)	3 (1.4)
1–3 months	69 (17.6)	8 (3.8)
3–6 months	93 (23.7).	6 (2.8)
6–12 months	97 (24.7).	32 (15)
12-24 months	76 (19.4)	78 (36.6)
>24 months	22 (5.6)	52 (24.4)
>36 months	0 (0)^*^	30 (14.1)
Unsure	3 (0.8)	4 (1.9)
Prefer not to answer	2 (0.5)	0 (0)
**What is the main reason that you started on PPPBF?**		
**(included only in the 1st Survey)**		
Allergy to one or more ingredients in other formulas	47 (12.0)	
Doctor or dietitian recommended	66 (16.8)	
Intolerance to one or more formulas used before	69 (17.6)	
Poor growth on previous formula	8 (2.0)	
Poor weight gain on previous formula	36 (9.2)	
Preferred ingredients in PPPBF	135 (34.4)	
Preferred the taste of PPPBF	7 (1.8)	
Other	24 (6.1)	
Unsure	1 (0.3)	

^*^Not included as a question in the 1st survey.

### Participant clinical diagnoses

Participants were asked for the main diagnosis that required a formula. They were only able to choose a single answer from those listed, which also included “other” (Table 3). The largest percentage of participants chose “other” in both surveys, 23% (*n* = 90) and 19.2% (*n* = 41) respectively. For the cohort from the 1st survey, the top five listed diagnoses were gastroparesis (*n* = 66, 16.8%), head and neck cancer (*n* = 34, 8.7%), difficulty swallowing from a disease not described (*n* = 34, 8.7%), failure to thrive (FTT) (*n* = 31, 7.9%), and malnutrition (*n* = 29, 7.4%). The top 5 diagnoses reported by the 2nd survey cohort included gastroparesis (*n* = 38, 17.8%) as the top diagnosis after “other”, followed by malnutrition (*n* = 21, 9.9%), FTT (*n* = 20, 9.4%), dysphagia (*n* = 19, 8.9%), and cerebral palsy (CP) (*n* = 13, 6.1%).

**Table 3:** Participant clinical diagnoses.

**Diagnosis**	**1st survey (*n* = 392) *n* (%)**	**2nd survey (*n* = 213) *n* (%)**
Gastroparesis	66 (17)	38 (17.8)
Dysphasia	34 (9)	19 (8.9)
Head and Neck Cancer	34 (9)	12 (5.6)
Failure to Thrive	31 (8)	20 (9.4)
Malnutrition	29 (7)	21 (9.9)
Other Cancer	25 (6)	8 (3.8)
Developmental Delay	16 (4)	8 (3.8)
Cerebral Palsy	22 (5)	13 (6.1)
Cystic Fibrosis	15 (4)	11 (5.2)
ALS	12 (3)	4 (1.9)
Brain Injury	7 (2)	5 (2.3)
Esophageal Cancer	7 (2)	1 (0.5)
Stroke	2 (0.5)	0 (0)
Achalasia	2 (0.5)	0 (0)
Other	90 (23)	41 (19.2)
Difficulty swallowing from a disease not described	34 (8.7)	10 (4.7)
Congenital heart disease	0 (0)	1 (0.5)

### Reported outcomes

Both surveys asked about the primary reason for stopping the PPPBF within the previous 12 months. If the formula user was still using the PPPBF at the time of the survey, they were able to select the answer “I am still using the PPPBF.” At the time of the 1st survey, 249 (63.5%) reported to still be using a pea protein plant based enteral formula; of those who reported to have discontinued the PPPBF formula, 49 (12.5%) no longer had a need for formula, and 17 (4.3%) reported to not tolerate the formula. Other reasons for discontinuing the use of the PPPBF included the lack of insurance coverage, 34 (8.7 %); expense, 16 (4.1%); taste, 4 (1.0%) and other, 23 (5.9%). At the time of the 2nd survey, 59.6% of participants (*n* = 127) reported to still be using the PPPBF. The highest reported reason for stopping the PPPBF was no longer having a need for formula (*n* = 35, 16.4%), followed by not having insurance coverage (*n* = 13, 6.1%).

In the 1st survey, almost 3/4 of the respondents, 278 (72.6%) reported that more than 50% of the PPPBF user's total nutrition was obtained through the formula, and 150 (38.2%) noted that the PPPBF provided > 90 %; a year later, at the time of the 2nd survey, slightly less, 29.6% of participants reported that more than 90% of their nutrition was from a PPPBF.

Questions about adherence were included in both surveys. The first survey, 40.1% of participants strongly agreed/agreed that they were able to use at least 75% of their previous formula. When asked about adherence to the PPPBF, 83.7% of participants strongly agreed/agreed they were able to use at least 75% of the recommended amount. In the 2^nd^ survey, 83.6% (*n* = 178) also strongly agreed/agreed to being able to use at least 75% of their PPPBF, mirroring the initial survey's adherence findings (Table 4).

**Table 4:** Reported adherence.

**While on the formula, I was able to use at least 75% (3/4) of the recommended amount**	**1st survey September 2019** ***n*** **(%)**		**2nd survey September 2020 *n* (%)**
	**Previous formula (non-PPPBF)**	**PPPBF**	**PPPBF**
Strongly agree/Agree	157 (40.1)	329 (83.7)	178 (83.5)
Strongly agree	83 (21.2%)	215 (54.7%)	104 (48.4%)
Agree	74 (18.9%)	114 (29%)	74 (34.7%)
Neutral	63 (16.1%)	22 (5.6%)	22 (10.3%)
Disagree	73 (18.6%)	19 (4.8%)	9 (4.2%)
Strongly disagree	52 (13.3%)	4 (1.0%)	3 (1.4%)
Prefer not to answer	47 (12%)	2 (0.5%)	1 (0.5%)

Both surveys included questions about reported weight change while using the PPPBF. In the 1st survey, weight change was reported by 58.3% (*n* = 229) of participants as weight gain and 6.4% (*n* = 25) as weight loss, while 27.7% (*n* = 109) reported no change in weight. In the 2nd survey, weight gain was reported by 64.3% (*n* = 137), 22.5% (*n* = 48) reported no weight change, and 7% (*n* = 15) weight loss while using the PPPBF.

In both surveys, participants were asked if they felt the PPPBF improved their digestive symptoms. A similar percentage of participants selected “strongly agree or agree” to this statement, 62.6% in the initial survey and 67.6% in the 2nd survey, 12-months later (Table 5).

**Table 5:** Reported improvements of digestive symptoms while using a PPPBF.

**The PPPBF improved my digestive symptoms (i.e.: easier bowel movements; less of any of the following: reflux, stomach aches, bloating, nausea, etc)**	**1st survey September 2019 *n* (%)**	**2nd survey September 2020 *n* (%)**
Strongly agree/Agree	246 (62.6)	144 (67.6)
Strongly agree	122 (31%)	68 (31.9%)
Agree	124 (31.6%)	76 (35.7%)
Neutral	109 (27.7%)	52 (24.4%)
Disagree	25 (6.4%)	14 (6.6%)
Strongly disagree	9 (2.3%)	3 (1.4%)
Prefer not to answer	4 (1.0%)	0 (0%)

A *post-hoc* analysis of the pediatric tube fed patients was completed to investigate the reported symptoms and adherence of those (*n* = 40) using a PPPBF formula. Comparisons were made to evaluate symptom frequency and adherence between the PPPBF formula and the previous formula. While those on the PPPBF had fewer episodes of each of the tracked symptoms when compared to their previous formula, the most significant changes were less bloating (*p* = 0.038) and less constipation (*p* = 0.04). Adherence was markedly better for the PPPBF (*p* < 0.001) compared to their previous formula, with 36 (97.3%) of those on the peptide (hydrolyzed) PPPBF able to take 75% of their recommended amount vs. 20 (55.6%) able to do the same on their previous formula. Previous formulas reported to be at least one of these types: standard intact dairy protein-based adult or pediatric specific formulations (44.9%), whey or casein peptide (hydrolyzed) protein-based adult or pediatric specific formulations (6.6%), infant formulas (4.8%), commercially available pediatric and adult blenderized formulations (7.4%), and amino acid/elemental protein-based pediatric formulations (5.4%), and “other” (45.9%). This analysis excluded those who did not respond or otherwise did not have a response for symptom frequency and adherence.

## Discussion

Enteral nutrition used as a tube feeding, or an oral nutrition supplement (ONS) should be well-tolerated to achieve a nutritional intake goal. Digestive symptoms such as diarrhea, vomiting, bloating, abdominal pain, and constipation can cause prolonged diminished nutrition intake and subsequently influence malnutrition-related side effects (9). This report of the use of a PPPBF over a sustained (12 month) period demonstrates a higher reported adherence and GI tolerance as compared to what was reported for the previously used enteral nutrition formulas. For those still using the PPPBF 12-months later, the reported improved digestive symptoms were similar to the initial survey report, suggesting a sustained benefit. Additionally, weight gain or stability were reported for 86.7 and 86.8% of respondents in the first and second surveys, respectively, with few (6.4% and 7 %) reporting weight loss in the two surveys. Although participants were not asked if the weight loss was intentional. Correlation with tolerance or adherence was outside the parameters of this investigation.

This study does not address the proposed mechanisms behind the improved tolerance that was reported in this cohort. Numerous studies have identified potential health benefits of plant-based diets often attributed to the nature of these diets being high in fiber and phytonutrients (15). PPPBF, in comparison to many dairy or amino acid-based enteral formulas, contains intact or partial hydrolyzed highly bioavailable yellow pea protein with mixed fiber (16).

Due to normal variations seen with different cultivars, growing conditions, and extraction methods, yellow pea protein (YPP), by itself, has a protein digestibility corrected amino acid score (PDCAAS) between 0.18 and 0.89, an *in vitro* digestibility between 83% and 95%, an *in vivo* digestibility of 98%. With the addition of limiting amino acids, the PPPBF in this study had a PDCAAS of 1.0. PDCAAS is the preferred method for measuring protein quality; it assesses the ability of a dietary protein to meet the body's amino acid requirements using both the digestibility and the amino acid score in the calculation (17–19). The PPPBF used in this study is made with an organically grown yellow pea protein which contains naturally occurring arginine and both soluble and insoluble fiber. Additionally, the PPPBF has a moderate rate of gastric digestion and is made without the top 9 US food allergens (20). Recent research has demonstrated that YPP has similar abilities to support muscle synthesis compared to whey protein (21, 22).

Cows' milk protein is the most common food allergen and recognized to affect gastrointestinal motility in children, contributing to constipation, a symptom of GI intolerance. No predictive and accurate biomarker for food allergy-associated constipation has been identified (23). The prevalence of food allergy underlying chronic constipation in children resistant to conventional treatment and presenting to tertiary clinics ranges between 28% and 78% (23). The avoidance of food allergens, such as cow's milk, soy and corn protein may be contributing to the reported improved digestive symptoms while on PPPBF of 62.6% and 67.6% of participants in the two survey groups.

PPPBF is made without artificial sweeteners, non-nutritive sweeteners, and artificial colors. In the United States, artificial and non-nutritive sweeteners have been a common ingredient in enteral formulas that contain hydrolyzed or amino acid-based protein sources. There is a growing body of literature linking non-nutritive sweetener intake to both an increase in functional GI disorder symptoms and an impact on gastric motility (24, 25).

While PPPBF contains a mix of insoluble and soluble fibers, the added organic agave inulin is a soluble and fermentable prebiotic fiber that may contribute to maintaining a healthy gut microbiome with minimal GI upset and improved laxation (26–28). Organic agave inulin is classified as a low viscosity fiber (29). Such fibers have been shown to exert minimal effects on mouth-to-cecum transit compared with higher viscosity soluble fibers (29). In this cohort, with the exception of “other,” gastroparesis was the most commonly reported primary diagnosis. Given that delayed gastric emptying can substantially influence gastrointestinal function and symptom burden, this finding underscores the clinical relevance of fiber type selection in this population. Furthermore, fermentable fibers have been shown to promote SCFA production, which in turn can improve colonic motility (30).

### Strengths and limitations of the study

Several limitations intrinsic to the study design must be considered when interpreting the findings. First, reliance on self-reported survey data introduces the potential for recall bias and subjective interpretation, which can affect the accuracy of reported outcomes such as GI tolerance, adherence, and weight changes. Participants may have unintentionally misreported symptoms or dietary adherence due to memory lapses or the desire to present themselves in a favorable light, thus potentially inflating perceived benefits or underreporting adverse experiences. Additionally, the lack of standardized definitions for GI intolerance required participants to rely on their own understanding, further complicating data consistency and comparability across responses.

Another limitation is the absence of a detailed review of previous formula types used by participants. While information on prior formula use was collected, the study did not include a systematic manual review of individual survey responses to track specific formula changes. Furthermore, the heterogeneity of the study population and the subjective nature of the survey responses reduce the generalizability and strength of the conclusions.

A strength of the study is that these results are consistent with a retrospective review on GI tolerance recently published (31). That study included 73 pediatric patients with health care providers in 17 different states with the majority specializing in gastroenterology (61.6%). They reviewed electronic medical records of 73 patients who transitioned from a hypoallergenic formula to a PPPBF and input de-identified patient information in Research Electronic Data Capture (REDcap). After the transition to the PPPBF, 38.4% of the patients experienced improvement in GI tolerance, 56.2% experienced no change in GI tolerance, and 5.5% reported worsening (31). Additionally, the percentages of responses to several questions were similar between both surveys, which may mean that those still using the PPPBF 12-months later, had sustained their initially reported level of adherence and improvement in digestive symptoms, or tolerance. Lastly, the surveys in this report are important to acknowledge as a valuable contribution to our understanding of patient and caregiver-reported outcomes while using a PPPBF, given that the literature on pea protein use in a clinical setting is limited.

### Clinical implications

This study provides clinicians and researchers with an important initial perspective on the clinical profiles and feeding routes of pediatric and adult patients who transitioned to an intact or peptide-based PPPBF. The significance of this study lies in its pioneering focus on pea protein plant-based formulas in medically complex patients requiring nutrition support; a population for whom evidence-based guidance is urgently needed. As the first investigation of its kind, it lays a foundation for future research and clinical practice, highlighting the necessity for rigorous, systematic evaluation of novel enteral nutrition options. While previously published articles assist in reducing formula switching non-specific to PPPBF enteral nutrition, a recent publication guides healthcare providers in selecting plant-based formulas (31). These promising findings and advancements in pea protein plant-based nutrition warrant further investigation through a prospective trial for validation. Of course, the aforementioned limitations intrinsic to the study design must be considered when interpreting the findings. To address these gaps, future research should incorporate objective data sources, specifically, utilizing electronic medical records to verify and document formula changes, allowing for precise tracking of nutritional interventions and outcomes. Additionally, gathering detailed information from both participants and healthcare providers regarding prior formula use and regimen recommendations would help clarify the influence of formula switching and support more nuanced analyses with stronger conclusions.

## Conclusion

Patients and caregivers reported responses to surveys a year apart, captured clinical and quality of life outcomes as they are perceived. Together, the findings highlight the critical impact of enteral nutrition (EN) formula selection on patient outcomes, emphasizing how the initial choice can influence GI tolerance, adherence, and lower the potential risk of malnutrition and its untoward effects.

## Data Availability

The raw data supporting the conclusions of this article will be made available by the authors, without undue reservation.

## References

[1] Doley J. Enteral nutrition overview. Nutrients. (2022) 14:2180. doi: 10.3390/nu14112180 35683980 PMC9183034

[2] A.S.P.E.N. Board of Directors and the Clinical Guidelines Task Force. Guidelines for the use of parenteral and enteral nutrition in adult and pediatric patients. JPEN J Parenter Enteral Nutr. (2002) 26:1SA−138SA. doi: 10.1177/0148607102026001011 11841046

[3] Malone A Carney LN Carrera AL Mays A editors. Chapter 2. In: AS*PEN Enteral Nutrition Handbook*. 2nd edn. Silver Spring, MD: American Society for Parenteral and Enteral Nutrition (2019). p.65–96.

[4] Eveleens RD Joosten KFM de Koning BAE . Definitions, predictors, and outcomes of feeding intolerance in critically ill children: a systematic review. Clin Nutr. (2020) 39:685–93. doi: 10.1016/j.clnu.2019.03.026 30962102

[5] Wischmeyer PE. Overcoming challenges to enteral nutrition delivery in critical care. Curr Opin Crit Care. (2021) 27:169–76. doi: 10.1097/MCC.0000000000000801 33395086 PMC8276753

[6] Barker L Gout B Crowe T. Hospital malnutrition: prevalence, identification, and impact on patients and the healthcare system. Int J Environ Res Public Health. (2011) 8:514–27. doi: 10.3390/ijerph8020514 21556200 PMC3084475

[7] Gramlich L Guenter P. Enteral nutrition in hospitalized adults. N Engl J Med. (2025) 392:1518–30. doi: 10.1056/NEJMra2406954 40239069

[8] Aloy Dos Santos T Luft VC Souza GC . Malnutrition screening tool and malnutrition universal screening tool as predictors of prolonged hospital stay and hospital mortality: a cohort study. Clin Nutr ESPEN. (2023) 54:430–5. doi: 10.1016/j.clnesp.2023.02.008 36963890

[9] Goates S Du K Braunschweig CA Arensberg B. Economic burden of disease-associated malnutrition at the state level. PLoS ONE. (2016) 11:e0161833. doi: 10.1371/journal.pone.0161833 27655372 PMC5031313

[10] Koontalay A Suksatan W Teranuch A. Early enteral nutrition met calories goals led by nurse on improve clinical outcome. Iran J Nurs Midwifery Res. (2021) 26:392. doi: 10.4103/ijnmr.IJNMR_421_20 34703776 PMC8491827

[11] Al-Beltagi M Saeed NK Bediwy AS Elbeltagi R. Cow's milk-induced gastrointestinal disorders: From infancy to adulthood. World J Clin Pediatr. (2022) 11:437–54. doi: 10.5409/wjcp.v11.i6.437 36439902 PMC9685681

[12] Flom JD Sicherer SH. Epidemiology of cow's milk allergy. Nutrients. (2019) 11:1051. doi: 10.3390/nu11051051 31083388 PMC6566637

[13] Warren CM Agrawal A Gandhi D Gupta RS. The US population-level burden of cow's milk allergy. World Allergy Organ J. (2022) 15:100644. doi: 10.1016/j.waojou.2022.100644 35539895 PMC9046619

[14] Patridge EF Bardyn TP. Research electronic data capture (REDCap). J Med Libr Assoc. (2018) 106:142–4. doi: 10.5195/jmla.2018.319

[15] Peña-Jorquera H Cid-Jofré V Landaeta-Díaz L Petermann-Rocha F Martorell M Zbinden-Foncea H . Plant-based nutrition: exploring health benefits for atherosclerosis, chronic diseases, and metabolic syndrome—a comprehensive review. Nutrients. (2023) 15:3244. doi: 10.3390/nu15143244 37513660 PMC10386413

[16] Verduci E Salvatore S Bresesti I Di Profio E Pendezza E Bosetti A . Semi-elemental and elemental formulas for enteral nutrition in infants and children with medical complexity—thinking about cow's milk allergy and beyond. Nutrients. (2021) 13:4230. doi: 10.3390/nu13124230 34959782 PMC8707725

[17] Rutherfurd SM Fanning AC Miller BJ Moughan PJ. Protein digestibility-corrected amino acid scores and digestible indispensable amino acid scores differentially describe protein quality in growing male rats. J Nutr. (2015) 145:372–9. doi: 10.3945/jn.114.195438 25644361

[18] Thavarajah D Lawrence T Boatwright L Windsor N Johnson N Kay J . Organic dry pea (*Pisum sativum* L.): a sustainable alternative pulse-based protein for human health. Plos ONE. (2023) 18:e0284380. doi: 10.1371/journal.pone.0284380 37043476 PMC10096185

[19] Shanthakumar P Klepacka J Bains A Chawla P Dhull SB Najda A. The current situation of pea protein and its application in the food industry. Molecules. (2022) 27:5354. doi: 10.3390/molecules27165354 36014591 PMC9412838

[20] Overduin J Guerin-Deremaux L Wils D Lambers TT. NUTRALYS^®^ pea protein: characterization of *in vitro* gastric digestion and *in vivo* gastrointestinal peptide responses relevant to satiety. Food Nutr Res. (2015) 59:1. doi: 10.3402/fnr.v59.25622 25882536 PMC4400298

[21] Pinckaers PJ Smeets JS Kouw IW Goessens JP Gijsen AP de Groot LC . Post-prandial muscle protein synthesis rates following the ingestion of pea-derived protein do not differ from ingesting an equivalent amount of milk-derived protein in healthy, young males. Eur J Nutr. (2024) 63:893–904. doi: 10.1007/s00394-023-03295-6 38228945 PMC10948472

[22] Babault N Païzis C Deley G Guérin-Deremaux L Saniez MH Lefranc-Millot C . Pea proteins oral supplementation promotes muscle thickness gains during resistance training: a double-blind, randomized, Placebo-controlled clinical trial vs. Whey protein. J Int Soc Sports Nutr. (2015) 12:3. doi: 10.1186/s12970-014-0064-5 25628520 PMC4307635

[23] Connor F Salvatore S D'Auria E . Cow's milk allergy-associated constipation: when to look for it? A narrative review. Nutrients. (2022) 14:1317. doi: 10.3390/nu14061317 35334974 PMC8955686

[24] Mendoza-Martínez VM Zavala-Solares MR Espinosa-Flores AJ . Is a non-caloric sweetener-free diet good to treat functional gastrointestinal disorder symptoms? A randomized controlled trial. Nutrients. (2022) 14:1095. doi: 10.3390/nu14051095 35268070 PMC8912523

[25] Bueno-Hernandez N Jimenez-Cruz BL Zavala-Solares M Melendez-Mier G. Association of natural and artificial nonnutritive sweeteners on gastrointestinal disorders: a narrative review. J Nutr Food Sci. (2018) 8:4. doi: 10.4172/2155-9600.1000711

[26] Holscher HD Doligale JL Bauer LL Gourineni V Pelkman CL Fahey GC . Gastrointestinal tolerance and utilization of agave inulin by healthy adults. Food Funct. (2014) 5:1142–9. doi: 10.1039/c3fo60666j 24664349

[27] Kennedy PJ Cryan JF Dinan TG Clarke G. Irritable bowel syndrome: a microbiome-gut-brain axis disorder? World J Gastroenterol. (2014) 20:14105–25. doi: 10.3748/wjg.v20.i39.14105 25339800 PMC4202342

[28] Liu L Zhu G. Gut-brain axis and mood disorder. Front Psychiatry. (2018) 9:223. doi: 10.3389/fpsyt.2018.00223 29896129 PMC5987167

[29] Suresh H Zhou J Ho V. The short-term effects and tolerability of low-viscosity soluble fibre on gastroparesis patients: a pilot clinical intervention study. Nutrients. (2021) 13:4298. doi: 10.3390/nu13124298 34959850 PMC8704257

[30] Fu J Zheng Y Gao Y Xu W. Dietary fiber intake and gut microbiota in human health. Microorganisms. (2022) 10:2507. doi: 10.3390/microorganisms10122507 36557760 PMC9787832

[31] Withrow NA Al-Tawil Y Patterson PJ Wilson M Ryan E Millovich V . Retrospective cohort study demonstrates tolerance and adherence to pea-based complete enteral formula when transitioned from a previous hypoallergenic product. Nutrients. (2024) 16:3365. doi: 10.3390/nu16193365 39408332 PMC11479067

