# Supplementary material for: Patient-reported outcomes on gastrointestinal tolerance and adherence to a pea protein plant-based enteral formula in children and adults
Source: Front Nutr. 2025 Dec 17;12:1619884. doi: 10.3389/fnut.2025.1619884 (PMC12755147; doi:10.3389/fnut.2025.1619884)
Supplement: Supplementary file 1 [file Table_1.docx]

Supplementary Material

**Figure 1. Survey 1 and 2**

**Survey 1**

1. What is your relationship to the person who has or who is currently using a Kate Farms formula?

*Parent*

*Sibling*

*Grandparent*

*Other family member*

*Other caregiver (non-family)*

*Self (I’m the one who has or is using a Kate Farms formula)*

*Prefer not to answer*

1. *What is the age of the person who has used or is currently using a Kate Farms formula?*

*1-5 years*

*6-12 years*

*13-19 years*

*20-40 years*

*41-60 years*

*>60 years*

*Prefer not to answer*

1. *What is the gender of the person who uses Kate Farms?*

*Female*

*Male*

*Prefer not to answer*

1. *What is the main diagnosis that causes this person to need a formula?(you can only check1)*

*Gastroparesis*

*Cystic Fibrosis*

*Malnutrition*

*Failure to thrive*

*Brain Injury*

*ALS*

*Cerebral Vascular Accident (Stroke)*

*Cerebral Palsy*

*Head and Neck Cancer*

*Achalasia*

*Other Cancer*

*Developmental delay*

*Difficulty swallowing from a disease not described above*

*Organ transplant*

*Congenital Heart disease*

*Other*

*Prefer not to answer*

1. *Which Kate Farms formula was/is used?(Please check all that apply)*

*Kate Farms Pediatric Standard 1.2*

*Kate Farms Pediatric Peptide 1.5*

*Kate Farms Standard 1.0*

*Kate Farms Peptide 1.5*

*Komplete (vanilla, chocolate, or coffee)*

*Prefer not to answer (1, 0.3%)*

1. *How was/is Kate Farms formula used each day? (Please check all that apply)*

*By mouth*

*With a syringe*

*Gravity bag*

*Feeding Pump*

*Unsure*

*Prefer not to answer*

1. *How much of the pereson’s total nutrition comes from or did come from Kate Farms each day? (please make your best guess)*

*<50%*

*50-90%*

*>90%*

*Unsure*

*Prefer not to answer*

1. *How long has this person been using Kate Farms formula?*

*< 1 month*

*1-3 months*

*3-6 months*

*6-12 months*

*12-24 months*

*Over 24 months*

*Unsure*

*Prefer not to answer*

1. *I felt healthier while on Kate Farms Formula.*

*Strongly Agree*

*Agree*

*Neutral*

*Disagree*

*Strongly Disagree*

*Prefer not to answer*

1. *Kate Farms formula improved by nutrition.*

*Strongly Agree*

*Agree*

*Neutral*

*Disagree*

*Strongly Disagree*

*Prefer not to answer*

1. *Kate Farms formula improved my digestive symptoms (i.e.: easier bowel movements; less of any of the following: reflux, stomach aches, bloating, nausea, etc)*

*Strongly Agree*

*Agree*

*Neutral*

*Disagree*

*Strongly Disagree*

*Prefer not to answer*

1. *If you had improved digestive symptoms after using Kate Farms formula, which of the following symptoms improved? (Please select all that apply)*

*Diarrhea*

*Constipation*

*Vomiting*

*Reflux*

*Bloating*

*Stomach aches*

*Nausea*

*I didn’t have improved digestive symptoms while on Kate Farms*

*Prefer not to answer*

1. *What is the main reason that you started Kate Farms formula?*

*Allergy to one or more ingredients in other formulas*

*Intolerance to one or more formulas used before*

*Preferred the taste of Kate Farms formula (*

*Preferred ingredients in Kate Farms*

*Poor growth on previous formula*

*Poor weight gain on previous formula*

*Doctor or Dietitian recommended*

*Other*

*Unsure*

*Prefer not to answer*

1. *Did your weight change while using Kate Farms formula?*

*Yes-increased (weight gain)*

*Yes-decreased (weight loss)*

*No change*

*Unsure*

*Prefer not to answer*

1. *While on Kate Farms, on average, how many episodes of abdominal bloating did you have, per week?*

*None*

*<1*

*1-3*

*4-6*

*>6*

*Unsure*

*Not applicable*

*Prefer not to answer*

1. *While on Kate Farms, on average, how many episodes of abdominal pain did you have, per week?*

*None*

*<1*

*1-3*

*4-6*

*>6*

*Unsure*

*Not applicable*

*Prefer not to answer*

1. *While on Kate Farms, on average, how many episodes of constipation did you have, per week?*

*None*

*<1*

*1-3*

*4-6*

*>6*

*Unsure*

*Not applicable*

*Prefer not to answer*

1. *While on Kate Farms, on average, how many episodes of diarrhea did you have, per week?*

*None*

*<1*

*1-3*

*4-6*

*>6*

*Unsure*

*Not applicable*

*Prefer not to answer*

1. *While on Kate Farms, on average, how many episodes of nausea did you have, per week?*

*None*

*<1*

*1-3*

*4-6*

*>6*

*Unsure*

*Not applicable*

*Prefer not to answer*

1. *While on Kate Farms, on average, how many episodes of reflux did you have, per week?*

*None*

*<1*

*1-3*

*4-6*

*>6*

*Unsure*

*Not applicable*

*Prefer not to answer*

1. *While on Kate Farms, on average, how many episodes of vomiting did you have, per week?*

*None*

*<1*

*1-3*

*4-6*

*>6*

*Unsure*

*Not applicable*

*Prefer not to answer*

1. *While on Kate Farms, I was able to use at least 75% (3/4) of the recommended amount.*

*Strongly Agree*

*Agree*

*Neutral*

*Disagree*

*Strongly Disagree*

*No specific amount was recommended*

*Prefer not to answer*

1. *While on the previous formula, on average, how many episodes of abdominal bloating did you have, per week?*

*<1*

*1-3*

*4-6*

*>6*

*Unsure*

*Not applicable*

*Prefer not to answer*

1. *While on the previous formula, on average, how many episodes of abdominal pain did you have, per week?*

*<1*

*1-3*

*4-6*

*>6*

*Unsure*

*Not applicable*

*Prefer not to answer*

1. *While on the previous formula, on average, how many episodes of constipation did you have, per week?*

*<1*

*1-3*

*4-6*

*>6*

*Unsure*

*Not applicable*

*Prefer not to answer*

1. *While on the previous formula, on average, how many episodes of diarrhea did you have, per week?*

*<1*

*1-3*

*4-6*

*>6*

*Unsure*

*Not applicable*

*Prefer not to answer*

1. *While on the previous formula, on average, how many episodes of nausea did you have, per week?*

*<1*

*1-3*

*4-6*

*>6*

*Unsure*

*Not applicable*

*Prefer not to answer*

1. *While on the previous formula, on average, how many episodes of reflux did you have, per week?*

*<1*

*1-3*

*4-6*

*>6*

*Unsure*

*Not applicable*

*Prefer not to answer*

1. *While on the previous formula, on average, how many episodes of vomiting did you have, per week?*

*<1*

*1-3*

*4-6*

*>6*

*Unsure*

*Not applicable*

*Prefer not to answer*

1. *While on the previous formula, I was able to use at least 75%(3/4) of the recommended amount.*

*Strongly Agree*

*Agree*

*Neutral*

*Disagree*

*Strongly Disagree*

*No specific amount was recommended*

*Prefer not to answer*

1. *Which formula(s) were you using right before you started Kate Farms?*

*Boost/Ensure*

*Pediasure/Boost Kid Essentials*

*Nutren/Jevity*

*Peptamen/Vital*

*Real Food Blends*

*Liquid Hope/Nourish/Compleat Organic Blends*

*Elecare/Neocate*

*Infant Formula*

*Other*

1. *If you stopped using Kate Farms formula, what was the main reason?*

*No insurance coverage*

*Didn’t tolerate it*

*Didn’t like the taste*

*Too expensive*

*I no longer have a need for formula*

*Other*

*I am still using Kate Farms*

**Survey 2**

1. *In the last 12 months, have you or the person you care for, used or tried a Kate Farms formula?*

*Yes*

*No*

1. *Are you still using a Kate Farms formula?*

*Yes*

*No*

1. *If you stopped using a Kate Farms formula within the last 12 months, what was the main reason?*

*No insurance coverage*

*Didn’t tolerate it*

*Didn’t like the taste*

*Too expensive*

*I no longer have a need for formula*

*Other*

*I am still using Kate Farms formula*

*Prefer not to answer*

1. *If you selected "Other", please briefly explain*
2. *What is your relationship to the person who has or who is currently using a Kate Farms formula?*

*Parent*

*Sibling*

*Grandparent*

*Other family member*

*Other caregiver (non-family)*

*Self (I’m the one who has or is using a Kate Farms formula)*

*Prefer not to answer*

1. *What is the age of the person who has used or is currently using a Kate Farms formula?*

*1-5 years*

*6-12 years*

*13-19 years*

*20-40 years*

*41-60 years*

*>60 years*

*Prefer not to answer*

# *What is the gender of the person who has used/is using a Kate Farms formula?*

*Female*

*Male*

*Prefer not to answer*

# *What is the main reason/diagnosis that you are using a Kate Farms formula?*

*Gastroparesis*

*Failure to Thrive*

*Malnutrition*

*Cystic Fibrosis*

*Brain Injury*

*ALS*

*Achalasia*

*Cerebral Palsy*

*Head and Neck cancer*

*Other cancer*

*Developmental Delay*

*Dysphagia*

*Difficulty swallowing from a disease not described above*

*Organ transplant*

*Esophageal cancer*

*Congenital heart disease*

*Other*

*Prefer not to answer*

1. *If you selected “Other”, please briefly explain*
2. *Which Kate Farms formula(s) have been used over the last 12 months? (please check all that apply)*

*Kate Farms Peptide 1.5 plain*

*Kate Farms Pediatric Peptide 1.5 vanilla*

*Kate Farms Standard 1.0 vanilla*

*Kate Farms Standard 1.0 chocolate*

*Kate Farms Pediatric Standard 1.2 vanilla*

*Kate Farms Komplete (vanilla, chocolate, coffee)*

*Prefer not to answer*

1. *Has your health or diagnosis changed over the past year?*

*No*

*Yes*

*Unsure*

*Prefer not to answer*

1. *How long has this person been using a Kate Farms formula?*

*<1 month*

*1-3 months*

*3-6 months*

*6-12 months*

*12-24 months*

*>24 months*

*>36 months*

*Unsure*

*Prefer not to answer*

# *How was/is Kate Farms formula used each day? (Please check all that apply)*

*Oral/By Mouth*

*Bolus with syringe*

*Gravity bag*

*Pump*

# *How much of the person's total nutrition comes from or did come from a Kate Farms formula each day? (please make your best guess)*

*<50%*

*50-90%*

*>90%*

*Unsure*

*Prefer not to answer*

1. *I feel/felt healthier while on a Kate Farms formula.*

*Strongly Agree*

*Agree*

*Neutral*

*Disagree*

*Strongly Disagree*

*Prefer not to answer*

1. *The Kate Farms formula improves/improved my nutrition.*

*Strongly Agree*

*Agree*

*Neutral*

*Disagree*

*Strongly Disagree*

*Prefer not to answer*

# *The Kate Farms formula improves/improved my digestive symptoms. (i.e., easier bowel movements; less of any of the following: reflux, stomach aches, bloating, nausea, etc.)*

*Strongly Agree*

*Agree*

*Neutral*

*Disagree*

*Strongly Disagree*

*Prefer not to answer*

# *If you had improved digestive symptoms after using a Kate Farms formula, which of the following symptoms improved? (Please select all that apply)*

*Diarrhea*

*Constipation*

*Vomiting*

*Reflux*

*Bloating*

*Stomach aches*

*Nausea*

*I didn’t have improved digestive symptoms while on Kate Farms*

*Prefer not to answer*

# *Did your weight change in the past year while using a Kate Farms formula?*

*Yes – increased (weight gain)*

*Yes – decreased (weight loss)*

*No change*

*Unsure*

*Prefer not to answer*

# *While on a Kate Farms formula, on average, how many episodes of abdominal bloating do/did you have each week?*

# *<1*

*1-3*

*4-6*

*>6*

*Unsure*

*Not applicable (n/a)*

*Prefer not to answer*

# *While on a Kate Farms formula, on average, how many episodes of abdominal pain do/did you have each week?*

*<1*

*1-3*

*4-6*

*>6*

*Unsure*

*Not applicable (n/a)*

*Prefer not to answer*

# *While on a Kate Farms formula, on average, how many bowel movements do/did you have per day?*

*1-2*

*3-4*

*5-6*

*>6*

*1 every other day*

*1 every 3-4 days*

*Once per week*

*Unsure*

*Prefer not to answer*

1. *Kate Farms formula was easily consumed.*

*Strongly agree*

*Agree*

*Neutral*

*Disagree*

*Strongly Disagree*

*Unsure*

*Prefer not to answer*

1. *If you selected "Other" please briefly explain*

# *If you had difficulty consuming Kate Farms formula, what was the main reason that made consumption difficult?*

*Nausea*

*Early feelings of fullness*

*Taste of the formula*

*Texture of the formula*

*Swallowing difficulties*

*Stomach pain*

*Other*

*Unsure*

*Prefer not to answer*

# *While using a Kate Farms formula, did you have any difficulty consuming it (whether orally or via feeding tube)?*

*Yes*

*No*

*Unsure*

*Prefer not to answer*

1. *While using Kate Farms formula, what was your usual consumption each day? (in # of cartons)*

*1*

*2*

*3*

*4*

*>4*

*Unsure*

*Prefer not to answer*

*Not applicable (n/a)*

# *If you were recommended to consume Kate Farms formula by a Doctor, Dietitian, or other Health Care Provider, how many cartons were recommended for you to consume each day?*

*1*

*2*

*3*

*4*

*>4*

*No specified amount*

*Unsure*

*Prefer not to answer*

*Not applicable (n/a)*

# *Was Kate Farms formula recommended to you by a Doctor, Dietitian, or other Health Care Provider?*

*Yes*

*No*

*Unsure*

*Prefer not to answer*

# *While on a Kate Farms formula, I was able to use at least 75% (3/4) of the recommended amount*

*Strongly Agree*

*Agree*

*Neutral*

*Disagree*

*Strongly Disagree*

*Prefer not to answer*

# *While on a Kate Farms formula, on average, how many episodes of vomiting do/did you have, per week?*

*<1*

*1-3*

*4-6*

*>6*

*Unsure*

*Not applicable (n/a)*

*Prefer not to answer*

# *While on a Kate Farms formula, on average, how many episodes of relux do/did you have, per week?*

*<1*

*1-3*

*4-6*

*>6*

*Unsure*

*Not applicable (n/a)*

*Prefer not to answer*

# *While on a Kate Farms formula, on average, how many episodes of nausea do/did you have, per week?*

*<1*

*1-3*

*4-6*

*>6*

*Unsure*

*Not applicable (n/a)*

*Prefer not to answer*

# *While on a Kate Farms formula, on average, how many episodes of diarrhea do/did you have, per week?*

*<1*

*1-3*

*4-6*

*>6*

*Unsure*

*Not applicable (n/a)*

*Prefer not to answer*

# *While on a Kate Farms formula, on average, how many episodes of constipation do/did you have, per week? (kf_epi_const)*

*<1*

*1-3*

*4-6*

*>6*

*Unsure*

*Not applicable (n/a)*

*Prefer not to answer*
